# A pragmatic approach to sonothrombolysis in acute ischaemic stroke: the Norwegian randomised controlled sonothrombolysis in acute stroke study (NOR-SASS)

**DOI:** 10.1186/s12883-015-0359-4

**Published:** 2015-07-11

**Authors:** Aliona Nacu, Christopher E. Kvistad, Nicola Logallo, Halvor Naess, Ulrike Waje-Andreassen, Anne Hege Aamodt, Ragnar Solhoff, Christian Lund, Håkon Tobro, Ole Morten Rønning, Rolf Salvesen, Titto T. Idicula, Lars Thomassen

**Affiliations:** 1 Department of Neurology, Haukeland University Hospital, N-5021 Bergen, Norway; 2 Department of Clinical Medicine, University of Bergen, Bergen, Norway; 3 Centre for age-related medicine, Stavanger University Hospital, Stavanger, Norway; 4 Department of Neurology, Oslo University Hospital, Oslo, Norway; 5 Department of Neurology, Arendal Hospital, Arendal, Norway; 6 Department of Neurology, Skien Hospital, Skien, Norway; 7 Department of Neuroly, Akershus University Hospital, Nordbyhagen, Norway; 8 Department of Neurology, Bodø Hospital, Bodo, Norway; 9 Department of Neurology, St. Olavs Hospital, Trondheim, Norway

**Keywords:** Randomised controlled trial, Acute ischemic stroke, Arterial occlusion, Contrast, Recanalisation, Outcome, Safety, Sonolysis, Sonothrombolysis, Thrombolysis, Transcranial ultrasound

## Abstract

**Background:**

Ultrasound accelerates thrombolysis with tPA (sonothrombolysis). Ultrasound in the absence of tPA also accelerates clot break-up (sonolysis). Adding intravenous gaseous microbubbles may potentiate the effect of ultrasound in both sonothrombolysis and sonolysis. The Norwegian Sonothrombolysis in Acute Stroke Study aims in a pragmatic approach to assess the effect and safety of contrast enhanced ultrasound treatment in unselected acute ischaemic stroke patients.

**Methods/Design:**

Acute ischaemic stroke patients ≥18 years, with or without visible arterial occlusion on computed tomography angiography (CTA) and treatable ≤ 4½ hours after symptom onset, are included in NOR-SASS. NOR-SASS is superimposed on a separate trial randomising patients with acute ischemic stroke to either tenecteplase or alteplase (The Norwegian Tenecteplase Stroke Trial NOR-TEST). The NOR-SASS trial has two arms: 1) the thrombolysis-arms (NOR-SASS A and B) includes patients given intravenous thrombolysis (tenecteplase or alteplase), and 2) the no-thrombolysis-arm (NOR-SASS C) includes patients with contraindications to thrombolysis. First step randomisation of NOR-SASS A is embedded in NOR-TEST as a 1:1 randomisation to either tenecteplase or alteplase. Second step NOR-SASS randomisation is 1:1 to either contrast enhanced sonothrombolysis (CEST) or sham CEST. Randomisation in NOR-SASS B (routine alteplase group) is 1:1 to either CEST or sham CEST. Randomisation of NOR-SASS C is 1:1 to either contrast enhanced sonolysis (CES) or sham CES. Ultrasound is given for one hour using a 2-MHz pulsed-wave diagnostic ultrasound probe. Microbubble contrast (SonoVue®) is given as a continuous infusion for ~30 min. Recanalisation is assessed at 60 min after start of CEST/CES. Magnetic resonance imaging and angiography is performed after 24 h of stroke onset. Primary study endpoints are 1) major neurological improvement measured with NIHSS score at 24 h and 2) favourable functional outcome defined as mRS 0–1 at 90 days.

**Discussion:**

NOR-SASS is the first randomised controlled trial designed to test the superiority of contrast enhanced ultrasound treatment given ≤4½ hours after stroke onset in an unselected acute ischaemic stroke population eligible or not eligible for intravenous thrombolysis, with or without a defined arterial occlusion on CTA. If a positive effect and safety can be proven, contrast enhanced ultrasound treatment will be an option for all acute ischaemic stroke patients. EudraCT No 201200032341; www.clinicaltrials.gov NCT01949961.

## Background

The common denominator of acute ischaemic stroke is a thromboembolic arterial occlusion. Intravenous (iv) thrombolysis with recombinant tissue plasminogen activator (tPA) is the only proven effective treatment for achieving arterial recanalisation and does improve outcome if given within 4½ hours from symptom onset [1]. However, recanalisation is achieved in only 30-40 % of patients who undergo thrombolysis [2]. As a result, many patients are left with a substantial brain damage, with high rates of disability and mortality [3].

Transcranial ultrasound has been shown in vitro and in vivo to accelerate thrombolysis [4–6] Ultrasound energy promotes motion of fluid around the thrombus (microstreaming) [7], stimulates arterial dilation, weakens fibrin cross-links, increases uptake and penetration of tPA and increases tPA concentration within the thrombus [8–11]. Ultrasound may thus promote tPA delivery despite stagnant flow near the occlusion.

In animal studies, ultrasound with frequencies from 27 kHz to 200 kHz increases the thrombolytic effect of iv thrombolysis [12]. However, low-frequency transcranial ultrasound (<1 MHz) carries an increased risk of opening the blood–brain barrier (BBB) [13, 14] and is also associated with productions of undesired standing waves and cavitation in the brain [15]. Both opening of the BBB and occurrence of standing waves during low frequency ultrasound insonation may therefore increase intracerebral bleedings when combined with iv thrombolysis [16, 17]. These results play an important role in designing a safer application of transcranial ultrasound in the brain [15].

In humans, ultrasound waves with a frequency of 2 MHz augment clot lysis [18] and recanalization when combined with iv thrombolysis (sonothrombolysis). Sonothrombolysis with pulsed-wave 2 MHz ultrasound is not associated with an increased risk of symptomatic intracerebral haemorrhage [19, 20], but is associated with a nearly 3-fold increased likelihood of complete recanalization and a 2-fold increased likelihood of functional independence at 3 months [19]. But even 2 MHz transcranial ultrasound with iv tPA has been shown to possibly increase the risk of symptomatic bleedings (TUCSON) [21, 22]. In particular low frequency (300 KHz) ultrasound seems to increase the risk of cerebral bleedings (TRUMBI) [16]. One randomised study with transcranial color-coded sonography (TCCS) using a 1.8-MHz Doppler ultrasound probe showed a beneficial effect on recanalisation and short term outcome in patients with middle cerebral artery main stem occlusion (MCA-M1), but with a tendency toward more severe haemorrhagic transformations in the ultrasound group [23]. At present, pulsed-wave 2 MHz ultrasound seems to be the safest approach for sonothrombolysis.

Adding gaseous microbubbles may increase the clot lysis with and without iv tPA both in vitro and in vivo [24]. Microbubbles potentiate the effect of ultrasound [25–27]. When exposed to ultrasound, microbubbles oscillate, expand or collapse producing stable cavitation (sustained bubble activity), which agitates the surrounding fluid, produces fluid jets that erode the thrombus surface, increases the surface area for thrombolytic action and accelerates lysis of clots [28–31]. Stable cavitation is associated with increased treatment effect [32], whereas inertial cavitation leads to microbubble destruction and decreases ultrasound mediated enhancement of sonothrombolysis [33]. The effect of ultrasound in sonothrombolysis and sonolysis are mainly influenced by three factors [24]: 1) the size of microbubbles which influences their resonant frequency [33]; 2) the increasing attenuation with increasing frequency which limits the depth of treatment at higher frequencies and 3) the increased risk of standing waves in the cerebral cavity at lower frequencies [34]. The use of lower ultrasound frequencies has the advantage of improving skull penetration and position tolerance due to insonation of a larger volume [24], but may increase the incidence of intracranial bleedings [16]. Higher ultrasound frequency (2 MHz) is therefore used in a clinical diagnostic setting. Adding microbubbles, i.e., iv ultrasound contrast, may induce further acceleration of ultrasound enhanced thrombolysis in acute ischaemic stroke patients, leading to a more complete recanalisation and better clinical outcome [4].

Contrast enhanced ultrasound insonation of thrombi in the absence of iv tPA has achieved high rates of recanalisation in animal models [35]. Repeated oscillations of the microbubbles around the clot cause an increase in local temperature due to the absorption of the energy from the radiated sound field and create further heating through viscous friction in the blood [36]. Microbubbles also penetrate the clot and make the tunnels in the fibrin matrix [24]. Experimental evidence thus indicates that ultrasound as such can accelerate clot break-up and lysis, both through mechanical (acoustic cavitation) and enzymatic mechanisms [28].

A high proportion of acute ischaemic patients are ineligible for iv tPA. The effect of ultrasound on the thrombus in the absence of tPA (sonolysis) is clinically not well studied, but transcranial 2 MHz ultrasound may have a lytic effect on the thrombus also without administration of tPA [22, 37–39]. This ultrasound effect is enhanced by echo contrast [31]. The same mechanisms as in the experimental studies may apply in humans, although the temperature effect may be negligible due to the ultrasound attenuation through the skull and tissue. It is nevertheless conceivable that contrast enhanced sonolysis (CES) may be more effective than ultrasound alone and may even have a neuroprotective role in acute ischaemic stroke patients [37, 40, 41]. Previous sonothrombolysis studies have focused on the subset of stroke patients eligible for thrombolysis. A number of patients have contraindications to thrombolysis and are left without a therapeutic option. No previous studies have systematically studied the effect of ultrasound in a general stroke population not eligible for iv thrombolysis. These subsets of patients are in desperate need of a treatment. NOR-SASS aims at testing a therapeutic option for these patients.

Alteplase has been used in previous sonothrombolysis studies. A growing body of evidence indicates that alteplase has a negative effect on the ischemic brain, including cytotoxicity and increased permeability of the blood brain barrier facilitating cerebral oedema [42]. An alternative thrombolytic therapy that might be easier and safer to administer could lead to wider acceptance and use of thrombolytic therapy [43] . Tenecteplase may be the drug of choice [44]. Tenecteplase is a modified tissue plasminogen activator that is more fibrin-specific, more resistant to plasminogen activator inhibitor (PAI), has a higher thrombolytic potency, with a longer half-life than alteplase and can therefore be administered as an intravenous bolus [43, 45]. No prior studies have investigated the combination of tenecteplase with contrast enhanced sonothrombolysis. NOR-SASS aims at testing the safety of tenecteplase in combination with contrast enhanced sonothrombolysis.

Previous sonothrombolysis studies were conducted in patients with an identified thrombus in major intracranial arteries [19, 46]. No previous studies have looked into the effect of ultrasound in minor stroke in the absence of a visible thrombus in the major intracranial arteries. Recanalisation rate following iv thrombolysis is higher with smaller thrombi in distal arteries [47]. It is therefore conceivable that ultrasound has even better effect on small, not visible peripheral arterial thrombi, on perforator thrombotic occlusions, or even thrombi in the microcirculation [35]. Several areas of peripheral vessels are, however, not accessible by ultrasound. Standard approaches never the less allow insonation of most of the clinically very relevant MCA-M3/M4, parts of the anterior and posterior cerebral vascular bed (transtemporal window) and the brainstem arteries (transforaminal window). Directing the ultrasound beam accurately onto hypothetical small peripheral occlusions includes a large degree of uncertainty, but a slight “fanning” movement of the probe increases the probability of covering the relevant region of interest.

Given the limited effect of iv thrombolysis and the logistic challenges in endovascular treatment, new therapeutic approaches for patients with acute ischaemic stroke are imperative. This protocol defines a pragmatic framework for routine ultrasound treatment in acute ischaemic stroke.

### Hypothesis

We hypothesise that in patients with acute ischaemic stroke eligible for intravenous thrombolysis, the clinical effect of contrast enhanced sonothrombolysis (CEST), given within 4½ hours from symptom onset is superior to that of intravenous thrombolysis alone. CEST is safe and effective in both proximal and distal thrombus, and may achieve higher recanalisation rates and better clinical outcome than intravenous thrombolysis alone.

We further hypothesise that in patients with acute ischaemic stroke not eligible for intravenous thrombolysis, the clinical effect of contrast enhanced sonolysis (CES) given within 4½ hours from symptom onset is superior to that of no specific treatment. CES is safe and effective in both proximal and distal thrombus, and may achieve higher recanalisation rates and better clinical outcome than no specific treatment.

### Aims of the study

The aims are to assess recanalisation, safety and clinical outcome of 1) CEST versus sham CEST in patients eligible for intravenous thrombolysis (NOR-SASS A and B) and 2) CES versus sham CES in patients not eligible for intravenous thrombolysis (NOR-SASS C).

## Design and methods

NOR-SASS is a multi-centre PROBE (prospective randomised open-label blinded endpoint) trial, designed to establish the superiority of contrast enhanced ultrasound treatment versus sham ultrasound treatment in consecutively admitted patients with acute ischaemic stroke. NOR-SASS is superimposed on a trial randomising patients with acute ischemic stroke to either tenecteplase or alteplase (The Norwegian Tenecteplase Stroke Trial; NOR-TEST; ClinicalTrials.gov NCT 01949948). NOR-SASS A includes patients willing to be randomised to either tenecteplase or alteplase in NOR-TEST, NOR-SASS B includes patients who did not give consent to be randomised and thus receive routine alteplase, and NOR-SASS C includes patients not eligible for iv thrombolysis.

NOR-SASS compares 1) ultrasound + microbubbles + randomised alteplase/tenecteplase versus sham ultrasound + sham microbubbles (plain saline) + alteplase/tenecteplase (NOR-SASS A) or 2) ultrasound + microbubbles + non-randomised routine alteplase versus sham ultrasound + sham microbubbles + alteplase (NOR-SASS B) and 3) ultrasound + microbubbles versus sham ultrasound + sham microbubbles (NOR-SASS C).

**Fig. 1:**
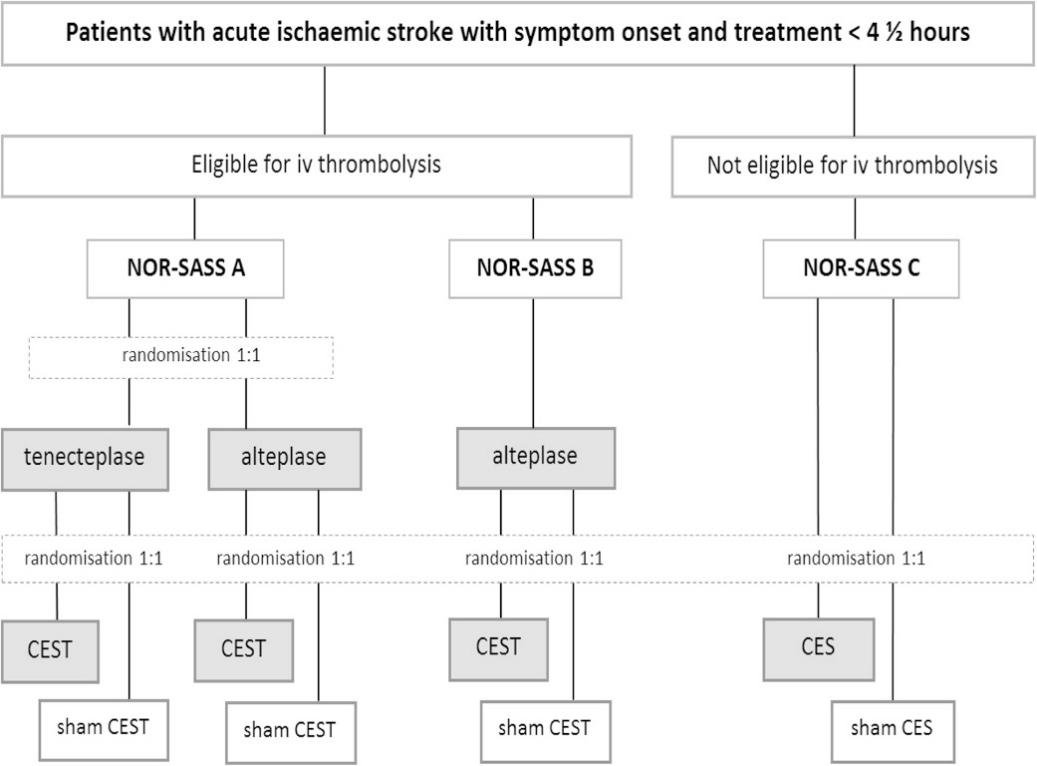
Randomization and flowchart. CEST Contrast enhanced sonothrombolysis with thrombolytic agent. CES Contrast enhanced sonolysis without thrombolytic agent

NOR-SASS A includes a first step randomisation 1:1 to either tenecteplase or alteplase and a second step randomisation 1:1 to either CEST or sham CEST. NOR-SASS B (alteplase group) randomisation is 1:1 to either CEST or sham CEST. NOR-SASS C randomisation is 1:1 to either CES or sham CES (Fig. 1).

### Power calculation

Our NORSTROKE data base from before the initiation of the present study shows that patients treated with thrombolysis (n = 223) had a 37 % improvement of mean NIHSS from 8.7 to 5.5 (3.2; SD = 5.9) from admission to day 7. Based also on thrombolysis series in mild stroke and on a meta-analysis of sonothrombolysis studies [48], NOR-SASS aims at detecting a 15 % higher reduction of mean NIHSS among patients receiving sonothrombolysis vs. thrombolysis alone (p_1_ = 0.37; p_2_ = 0.52, power 0.8) and will include 276 patients over 3–4 years. The study was initiated at Haukeland University Hospital, Bergen, in Q3 2012 and is planned to close Q4 2016. An interim analysis is planned for Q2 2015. Per January 2014, 176 patients are enrolled.

### Patient inclusion and exclusion criteria

All acute ischaemic stroke patients ≥ 18 years admitted within 4½ hours of symptom onset may be included in the study. An intracranial arterial occlusion on pre-treatment computed tomography angiography (CTA) is not a prerequisite for inclusion since this study also includes sonothrombolysis/sonolysis of the peripheral vascular bed and central perforating arteries. Study participation is based on informed consent requirements as approved by the ethics committee. Exclusion criteria are listed in Table 1.

**Table 1:** Inclusion and exclusion criteria

Inclusion criteria
•Patients >18 years with acute ischaemic stroke, with or without a visible arterial occlusion, and start of treatment within 4 ½ hours after stroke onset.
General exclusion criteria
•Patients with premorbid modified Rankin Scale (mRS) score ≥3
•Patients for whom a complete NIH Stroke Score cannot be obtained
•Hemiplegic migraine with no arterial occlusion on baseline CT
•Seizure at stroke onset and no visible occlusion on baseline CT
•Intracranial haemorrhage on baseline CT
•Clinical presentation suggesting subarachnoid haemorrhage even if baseline CT is normal
•Large areas of hypodense ischaemic changes on baseline CT
Patients with primary endovascular treatment
•Pregnancy or breast feeding, pericarditis, sepsis, any other serious medical illness likely to interact with treatment, confounding pre-existent neurological or psychiatric disease, unlikely to complete follow-up, any investigational drug <14 days
Specific sonothrombolysis/sonolysis exclusion criteria
•Known hypersensitivity or allergy to SonoVue®
•Recent or unstable coronary ischemia or resting angina <7 days
•Acute cardiac insufficiency, cardiac insufficiency class III/IV; serious cardiac arrhythmias
•Any right-left-shunt, severe pulmonary hypertension (PAP >90 mmHg) Moderate to severe chronic obstructive pulmonary disease (chronic obstructive pulmonary disease (COPD), baseline O2 saturation <80 %)
•Acute respiratory distress syndrome (ARDS)

### Study endpoints

Primary study endpoints are 1) neurological improvement (NIHSS score) at 24 h (clinical effect; proof of concept) and 2) functional handicap defined as modified Rankin Scale (mRS) score 0–1 at 90 days (clinical outcome). Secondary study endpoints are 1) haemorrhagic transformation (haemorrhagic infarct/haematoma), 2) symptomatic cerebral haemorrhage (safety endpoint), 3) recanalisation at 2 h (optional), 4) recanalisation at 24–36 h, 5) MR-DWI lesion volume at 24–36 h and 6) death.

### Assessment methods

Table 2 neurological deficit is documented with National Institutes of Health Stroke Scale (NIHSS). Function is documented with mRS and Barthel Index (BI). On admission, native computed tomography (CT) with CTA is performed for all patients within the therapeutic time window. Early CT ischaemic signs are semi-quantitatively assessed using the ASPECTS scoring system [49]. Intracranial middle cerebral artery (MCA) occlusions are trichotomised in none, distal (MCA_2_), or proximal (MCA_1_) occlusion. Other occlusions are defined as appropriate. Intracranial artery stenosis are described as minor (≤50 %), moderate (51-70 %) and severe (71-99 %) diameter reduction or occlusion. Magnetic resonance imaging and angiography (MRI/MRA) or CT/CTA is performed after 22–36 h to verify the infarct, assess intracranial recanalisation and to detect haemorrhagic transformation. Radiological assessment is performed by investigators blinded to the treatment given. Duplex ultrasound examinations are carried out the day after admission. Carotid stenosis grading is based on flow velocities calculated with a modified NASCET formula or with the internal-common carotid ratio (IC-CC ratio).

**Table 2:** Assessment of patients with acute ischaemic stroke admitted ≤4½ hours

Procedure ↓	Timepoint→	Baseline	1-2 h	+24 h (22-36 h)	+48 h (42–54 h)	Day 7 or earlier discharge	Day 90
NIHSS score		X	X	X	X	X	(X)
CT / CTA (MRI / MRA)		X		X			
ECG		X					
Duplex ultrasound of the neck				X			
Transcranial duplex ultrasound		X	X	(X)		(X)	
Modified Rankin Scale (mRS)		X				X	X
Barthel Index (BI)						X	(X)
Check Recurrent stroke / TIA							X
Check Acute coronary heart disease							X

*(X)* optional

The amount of ultrasound delivery to the vasculature cannot be assessed in NOR-SASS. As a surrogate marker for attenuation through the skull, bone window thickness is measured by a physician blinded to clinical information, except for the window of insonation.

### Long-term follow-up

Long-term follow-up is performed by standardised questionnaire as an outpatient clinic visit by a neurologist or as a telephone interview by a study nurse at 3 months. Modified Rankin Score, recurrent vascular events, other diseases or complications, employment state, marriage state, and mood are registered. Death is checked via the Norwegian Public Death Registry.

### Treatment

Patients are treated according to the Department’s Standard Operating Procedure (SOP). Specific intravenous or intra-arterial acute treatment, neurosurgery, and general medical treatment are registered in Norwegian Stroke Research Registry (NORSTROKE). Thrombolytic treatment must start ≤4½ hours after acute ischaemic stroke symptoms onset. Alteplase is given in a dose of 0.9 mg/kg (10 % bolus + 90 % infusion during 1 h), maximum dose 90 mg. Tenecteplase is given in a dose of 0.4 mg/kg as bolus, maximum dose 40 mg.

### Treatment with ultrasound and microbubbles (sonothrombolysis or sonolysis)

In patients without an angiographically defined clot, the investigator judges the clinical picture and applies ultrasound to the region in which she/he thinks the lesion/clot is located, either through the temporal or foraminal window as appropriate. In patients with a defined occlusion, ultrasound is applied to the region of the occlusion. All patients are examined and monitored with transcranial color-coded duplex sonography (TCCS) and transcranial doppler (TCD) performed by an experienced ultrasonographer working independently from the treating physician. Intravenous microbubbles (SonoVue®; 10 ml = 80 μl = 45 μg microbubbles) are given as an infusion of 0.3 ml/min for ~30 min, using a BraccoVueJet® infusion pump (BRACCO Imaging, Switzerland).

1. Baseline TCCS examination is performed to locate the best insonation window, to locate any occlusion and to assess haemodynamics. A temporal bone window is defined as good when it allows good visualisation of standard landmarks such as the mesencephalon. A foraminal window is defined as good when it allows good visualisation of the basilar artery. Bilateral assessment of MCA flow velocities may be undertaken to calculate inter-hemispheric asymmetry indices [50].
2. Sonothrombolysis or sonolysis is performed with 2 MHz pulsed wave TCD monitoring for 60 min, using either a hand-held TCD probe or a probe in a fixation head-band. TCD emitted power output is set at the maximal achievable level with mechanical index MI <1.0. The sound of TCD is muted and the visual display is turned away from the treating physicians and nurse in order to keep them blinded to the patients’ study-group assignment [8, 51]. Insonation is performed with continuous slight “fanning” of the presumed region of interest, with or without the head band.
3. Recanalisation is assessed at 60 min after start of sonothrombolysis or sonolysis with TCCS.

### Treatment with sham sonothrombolysis or sham sonolysis

After baseline diagnostic TCCS, the 2 MHz TCD probe is plugged into an inactive channel. Recanalisation is assessed using active TCCS at 60 min after start of sham sonothrombolysis or sonolysis. Sham intravenous contrast (NaCl 0.9 %) is given as an iv infusion of 0.3 ml/min for ~30 min, using a BraccoVueJet® infusion pump. Care is taken not to display to the treating physicians which infusion is given.

### Outcomes measures

#### Clinical outcome (neurology and function)

Early (2 h and 24–36 h) clinical outcome, short term (day 7, or earlier if earlier discharge) and long term (day 90) functional outcome are assessed in a blinded fashion by a trained stroke nurse or neurologist.

Early clinical outcome is defined by NIHSS score as 1) absolute reduction in NIHSS at 2 h (∆NIHSS_2_ = NIHSS_0_–NIHSS_2_) and at 24 h (∆NIHSS_24_ = NIHSS_0_–NIHSS_24_) and as 2) neurological improvement at 24 h (NIHSS_24_ = 0 or reduction of ≥4 NIHSS points compared with baseline NIHSS_0_). Short term outcome is defined by mRS score using sliding dichotomy/responder analysis (excellent outcome mRS 0 with baseline NIHSS ≤7, mRS 0–1 with baseline NIHSS 8–14, mRS 0–2 with baseline NIHSS ≥15).

Long term outcome is defined by mRS score using 1) single mRS group comparison; 2) fixed dichotomy (excellent = mRS 0–1 vs. unfavourable = mRS 2–6 outcome, and good = mRS 0–2 vs. bad mRS 3–6 outcome); and 3) sliding dichotomy/responder analysis (excellent outcome mRS 0 with baseline NIHSS ≤7, mRS 0–1 with baseline NIHSS 8–14, mRS 0–2 with baseline NIHSS ≥15).

### Vascular outcome (recanalisation)

In patients with a defined arterial occlusion on pre-treatment CTA, early recanalisation is assessed by TCCS at 60 min. Flow is categorised according to the Thrombolysis in Brain Ischaemia (TIBI) grading [52] modified as 0 = absent flow; 1 = minimal flow; 2 = reduced (TIBI 2–3 blunted/dampened) flow; 3 = restored (TIBI 4–5 stenotic/normal) flow.

In patients without a defined arterial occlusion on pre-treatment CTA, haemodynamics are assessed by TCCS at 60 min. In patients with MCA occlusions, inter-hemispheric asymmetry is calculated comparing time averaged mean flow velocities. Significant peripheral MCA obstruction(s) is defined as MCA_1_ mean flow velocities >21 % lower than in the contra-lateral MCA_1_ [50].

Recanalisation at 1 h is by necessity performed unblinded by the investigator. Recanalisation at 24-h is assessed by 24-h MRA (or CTA when MRA is not possible) by a neurologist and/or neuroradiologist blinded to all clinical data [53]. Vascular patency is assessed using adapted Thrombolysis in Myocardial Infarction (TIMI) criteria [54]. Vessel occlusion status is defined as complete occlusion (TIMI 0), minimal flow (TIMI 1), partial flow (TIMI 2), or normal flow (TIMI 3). Major vessel recanalisation is defined as improvement in baseline-24 h TIMI score of ≥2, or complete recanalisation.

### Brain tissue outcome (infarction)

Tissue damage is assessed by 24-h MRI diffusion weighted imaging (DWI) or (CT when MRI is not possible) by a neurologist and/or neuroradiologist blinded to all clinical data, using ASPECTS scoring system [49, 55]. In patients without a visible occlusion on initial CTA the MRI localisation of the lesion is compared with the clinically suspected localisation and will confirm whether the initial ultrasound was aimed at the correct region of interest or not.

### Safety outcome

Patients are monitored in the stroke unit (or intensive care unit (ICU) if appropriate) with NIHSS scoring at close intervals for 24–36 h. Safety is determined by the incidence of haemorrhagic transformation within 24-h of treatment on MRI (or CT when MRI is not possible) by a neurologist and/or neuroradiologist blinded to all clinical data. Classification of haemorrhagic transformation is left to the discretion of the local investigators. In case of disagreement, the scans are assessed by the Data Safety Monitoring Committee.

Haemorrhagic transformation (ECASS criteria) are categorised as *haemorrhagic infarction* 1 (HI1 = small petechiae along the margins of the infarct); *haemorrhagic infarction* 2 (HI2 = confluent petechiae within the infarcted area but no space-occupying effect); parenchymal haemorrhage 1 (*PH1* = blood clots in 30 % or less of the infarcted area with some slight space-occupying effect); p*arenchymal haemorrhage 2 (PH2* = blood clots in more than 30 % of the infarcted area with substantial space-occupying effect); and *Remote parenchymal haemorrhage (rPH =* bleeding outside the infracted area).

Symptomatic cerebral haemorrhage (sICH) is defined as local or remote parenchymal haemorrhage type 2 on the 22–36 h post-treatment imaging scan, combined with a neurological deterioration of 4 points or more on the NIHSS from baseline or from the lowest NIHSS value between baseline and 24 h, or leading to death *(SITS-MOST criteria)* [56]. In order to facilitate comparison with published studies, sICH is also assessed according to 1) NINDS /Cochrane criteria [57] as any haemorrhage plus any neurological deterioration [NIHSS score ≥1] or that leads to death within 7 days; 2) ECASS II criteria [58] as blood at any site in the brain on the CT scan, clinical deterioration or adverse events indicating clinical worsening (drowsiness, increase of hemiparesis) or causing an increase in the NIHSS score of 4 or more points; and 3) ECASS III criteria [59] as any apparently extravascular blood in the brain or within the cranium associated with clinical deterioration (defined by an increase in the NIHSS score of 4 or more points) or death, and that is identified as the predominant cause of the neurological deterioration.

### Safety monitoring

The over-all rate of bleeding complications will be compared with historical data from the NORSTROKE Registry 2007–2011, the Pooled analysis of iv alteplase trials [57], SITS-MOST [60, 61], ECASS III [59] and series on thrombolysis in patients above 80 years of age [62]. The mortality rate at 7 days will be compared with historical data from NORSTROKE 2007–2011.

All Suspected Unexpected Serious Adverse Reactions (*SUSARs)* are reported to the Norwegian Medicines Agency. All serious adverse events (SAE), i.e., bleedings and deaths, are reported within 24 h to the Executive working group (EWG). The EWG routinely submits a safety report to the independent Data Safety Monitoring Committee (DSMC) after each 40 enrolled patients (20 per group). If the DSMC finds unacceptably increased sICH with CEST+ thrombolysis as compared with sham CEST+ thrombolysis, the study may be stopped for further analysis. If CEST+ tenecteplase shows a 2 % excess of sICH as compared with CEST+ alteplase, the tenecteplase tier may be stopped and the study may be continued with only alteplase patients. The same stopping rule is applied to CES vs. sham CES in patients not eligible for iv thrombolysis.

### Statistical analysis

Statistical design is made in co-operation with the Centre for Clinical Research, Haukeland University Hospital. An interim analysis will be performed in Q2 2015. The exact method of the final analysis will be specified in the Statistical Analysis Plan, which will be drawn up prior to the closure of the database and the statistical analysis. The primary analysis is an “intention to treat” analysis. The results will also be tested in a “per protocol” analysis. Stratification according to age (≤80 versus >80), baseline NIHSS (≤14 versus >14), time (0–3 h vs. 3-4½ h), occlusion/no occlusion; correct/incorrect insonation focus (patients with no visible occlusion on CTA); atrial fibrillation/no atrial fibrillation will be performed. An analysis excluding MCA_1_ occlusion and MCA_1_- MCA_2_ occlusions will also be performed. Temporal bone window thickness will be included in the analysis. Serious Adverse Events (SAE) and Adverse Events (AE) will be analysed as appropriate.

### Organisation

This study is one of several studies under the Norwegian Stroke Research Co-operation (NORSTROKE) umbrella. NOR-SASS is an investigator driven academic trial with no bindings to pharmaceutical companies. The trial is independently monitored by the Western Norway Health Trust. Patient data are stored as a *NORSTROKE dataset* in the European Cerebrovascular Research Infrastructure (*ECRI) database* (Oslo University Hospital). The study is supported by the University of Bergen, the Norwegian Research Council and the Western Norway Health Trust.

### Approvals

NOR-SASS is performed in accordance with the Declaration of Helsinki, is approved by the Regional Ethics Committee and the Norwegian Medicines Agency, and is registered with EudraCT No 201200032341 and in www.clinicaltrials.gov with Identifier NCT01949961. The trial is conducted according to Good Clinical Practice: Consolidated guideline (CPMP/ICH/135/95).

## Discussion

Acute ischaemic stroke is a sudden disturbance of the blood supply to the brain, caused by a blood clot, which rapidly leads to severe brain damage. Rapid restauration of blood flow in the occluded intracerebral arteries is the main goal of acute ischaemic stroke treatment [39]. Rapid reopening of big or small occluded vessels with regional reperfusions and salvage of brain tissue, is strongly associated with improved clinical outcome and reduced mortality [63]. Despite many attempts to find a new treatment for acute ischaemic stroke in the last years, iv trombolysis remain the only effective treatment, but confers clinical benefit in only a limited number of patients [64]. A new or improved acute treatment is urgently needed.

Contrast enhanced ultrasound treatment, i.e., sonothrombolysis (with iv thrombolysis) and sonolysis (without iv thrombolysis), may be the new therapeutic option for patients with acute ischaemic stroke.

The CLOTBUST trial (Combined Lysis of Thrombus in Brain Ischemia Using Transcranial Ultrasound and Systemic tPA) was a phase II, multicenter international randomized clinical trial that compared iv tPA vs. iv tPA plus 2-MHz transcranial Doppler monitoring for 2 h in acute ischaemic stroke patients with middle cerebral artery occlusion, within a 3 h time window. A significant clinical recovery and complete reperfusion of the occluded artery was noted in 49 % of the patients treated with iv tPA and ultrasound vs. 30 % of the iv tPA alone, without an increase of intracerebral bleedings [8].

Lower frequency ultrasound is theoretically more effective than higher frequency diagnostic ultrasound, because of better penetration through the skull bone windows and stronger thrombolytic effect [65]. The TRUMBI trial (Transcranial Low-Frequency Ultrasound-Mediated Thrombolysis in Brain Ischemia) was a phase II, prospective, multicenter nonrandomized trial that compared standard iv tPA vs. tPA plus low-frequency pulsed-wave transcranial ultrasound (300-kHz) in patients with defined arterial occlusion within a six hour time window. The study was stopped prematurely because of an unexpected high rate of intracerebral bleeding in the group treated with iv tPA and low frequency ultrasound [16]. A technical study which simulated TRUMBI ultrasound delivery, demonstrated that the peak rarefactional pressure was higher than the inertial acoustic cavitation threshold in the presence of standing wave in large areas of the brain, even outside the targeted clot [66] . The technical setting in TRUMBI may therefore have contributed to increased intracerebral bleedings in the ultrasound group.

The TUCSON study (Transcranial Ultrasound in Clinical Sonolysis for acute ischaemic stroke) was a phase I-II, randomised, placebo-controlled, open label, safety, dose-escalation clinical trial which compared iv tPA vs. iv tPA plus 2 MHz ultrasound plus microbubbles in patients with documented intracranial arterial occlusion. The study showed a trend toward higher early recanalisation rate and clinical recovery, but was stopped prematurely by the sponsor, because three patients died in the group treated with iv tPA plus ultrasound plus microbubbles, indicating a possibly increased risk of symptomatic bleedings with this combination [22].

Despite TRUMBI and TUCSON trials results, one experimental animal model of intracerebral hemorrhage showed that ultrasound in combination with microbubbles did not cause additional brain damage during intracerebral hemorrhage [67]. The size of brain hemorrhage, the amount of apoptosis and extension of brain edema was similar between the ultrasound plus microbubbles group and the control group [67]. This was the first experimental study to show that the combination of ultrasound plus microbubbles is safe in cerebral bleedings and may be used in sonothrombolysis and sonolysis studies, even before conventional stroke imaging.

Also, a systematic review and meta-analysis of randomised controlled trials and case–control studies [68] from 1970 till 2013, which included 7 randomised control trials and 3 case control studies showed that sonothrombolysis and sonolysis is safe, effective (OR of complete recanalization (CR) at 1–2 h: 2.95;95 % CI: 1.81-4.81; *P* < 0.00001) and patients treated have more than two-fold higher likelihood of favourable long-term outcome (3-months mRS 0–2; OR: 2.20; CI:1.52-3.19; *P* < 0.0001). These findings are supported by earlier meta-analyses [19, 46]. Use of microbubbles in sonothrombolysis or sonolysis is safe and effective (OR of CR: 2.61; CI: 1.36-4.99; *P* = 0.004) [68].

NOR-SASS is the first randomised controlled phase III trial designed to test the superiority of contrast enhanced ultrasound treatment given ≤4½ hours after stroke onset in a general acute ischaemic stroke population eligible or not eligible for iv thrombolysis, and with or without a defined arterial occlusion on CTA. The primary aim of NOR-SASS study is to investigate the effect of a thrombolytic drug, 2 MHz ultrasound and microbubbles in all acute ischaemic patients.

Only 10 % of all acute ischaemic stroke patients within 4.5 h’ time window have a defined occlusion on CTA in The Bergen NORSTROKE Registry. At present, there are no data on the effect of sonothrombolysis or sonolysis in patients with less severe stroke in the absence of a visible thrombus. However, there is no reason to believe that contrast enhanced ultrasound treatment has inferior effect on small, not visible thrombi. This group consists of 90 % of all acute ischaemic stroke patients, but has not been included in previous sonothrombolysis and sonolysis clinical trials. The most prominent treatment effect with complete reversal of microcirculatory obstructions has been seen for contrast enhanced sonothrombolysis (tPA, ultrasound and microbubbles) vs. tPA with unenhanced ultrasound [35]. Also, a tissue protective effect and improved microcirculation has been observed with the combination of ultrasound and microbubbles [39].

NOR-SASS will add relevant new information on sonothrombolysis and sonolysis as a treatment for all acute ischaemic stroke patients within 4.5 h’ time window, with or without a defined arterial occlusion on CTA and not only in selected patients. Thrombolysis with alteplase (Actilyse®), transcranial ultrasound (TCCS, TCD) and ultrasound contrast (SonoVue®) are part of routine treatment and assessment. NOR-SASS differs from routine treatment through the combination of different kinds of thrombolytic drugs plus ultrasound plus microbubbles.

NOR-SASS includes tenecteplase (Metalyse®), an approved safe and effective drug for acute myocardial infarction, but so far not studied or approved in acute ischaemic stroke. NOR-SASS is the first sonothrombolysis study with tenecteplase. This alternative thrombolytic therapy, which is easy to use, safe to administer and has little toxicity may be a new choice for acute ischaemic stroke treatment with or without ultrasound. The main aim of NOR-SASS is, however, to study sonothrombolysis in patients receiving “any kind of thrombolysis”. The number of patients receiving tenecteplase will probably be too low to yield definite effect results, but is considered to be a phase II safety sub-study.

Microbubbles have their own ability to increase clot lysis in combination with ultrasound, even in the absence of a thrombolytic drug [69]. In an animal study, treatment with microbubbles and transcranial ultrasound without tPA (sonolysis) resulted in the same improvement as treatment with iv tPA alone and was associated with significant improvement compared with untreated animals [35]. This indicates that microbubbles with ultrasound may have a positive effect similar to that of iv tPA also in humans. Acute ischaemic stroke patients with contraindication for iv tPA within a 4.5 h time window, now untreated and “neglected”, may benefit from sonolysis and have the same clinical effect and outcome as after iv tPA. NOR-SASS C is the first randomised sonolysis study in all patients with acute ischaemic stroke that cannot receive thrombolysis. Even if the outcome improvement is moderate, sonolysis may still have a clinically relevant impact in this group.

### Thrombolysis in mild stroke

Mild strokes tend to resolve on their own relatively frequently. This will make it more difficult to detect a meaningful difference between the treatment groups. Own data indicate that even in mild stroke, thrombolysis improves outcome [70]. Also, most patients with mild stroke do not have a visible clot. Stroke mimics mostly present with mild symptoms, often seemingly related to the vertebrobasilar circulation. Including mild strokes in the study therefore probably increases the number of stroke mimics, which also makes it more difficult to detect a meaningful difference between the treatment groups. NOR-SASS aims, however, at testing sonothrombolysis in all acute stroke patients and by pragmatic necessity, NOR-SASS therefore includes also mild strokes, albeit with a risk of weakening the study.

### Delivery of ultrasound to the vasculature

Any stroke trial treatment should ideally be given with a defined dose. Transcranial ultrasound is attenuated through the skull and this reduces the effect of ultrasound. The attenuation varies between patients and the “dose” of ultrasound applied in sonothrombolysis is therefore not standardised. NOR-SASS is a clinical pragmatic study and does not include acoustic measurements to assess the effect of attenuation. Measurements of skull thickness in the area of insonation may, however, serve as a surrogate marker for skull penetration and thereby also the dose of ultrasound applied. In patients without a visualised clot, the investigator judges the clinical picture in cooperation with the treating physician and decides where she/he thinks the probable lesion/clot/ischemia may be. Ultrasound is applied based on this assumption. MR examination the next day demonstrates the DWI lesion(s). The final analysis thereby can include whether or not the initial ultrasound was aimed at the correct region of interest or not.

## Conclusion

Experimental and human literature supports the view that contrast enhanced sonothrombolysis and contrast enhanced sonolysis improves recanalisation and reperfusion, that it may save tissue at risk, without posing a threat to the patient’s well-being. If a positive effect and safety can be proven by NOR-SASS, contrast enhanced ultrasound treatment may be a pragmatic option for acute ischaemic stroke patients eligible or not eligible for intravenous thrombolysis, for patients with or without a defined arterial occlusion on CTA and for those who do not have access to intra-arterial treatment. And all evidence points in the same direction: sonothrombolysis and sonolysis represent the next step in non-invasive treatment of acute ischaemic stroke.

## Abbreviations

AE: Adverse events
ARDS: Acute respiratory distress syndrome
ASPECTS: Alberta stroke program early ct score
BI: Barthel index
BSRG: Bergen stroke research group
CES: Contrast enhanced sonolysis
CEST: Contrast enhanced sonothrombolysis
CI: Confidence interval
CIOMS: Council for international organizations of medical sciences
COPD: Chronic obstructive pulmonary disease
CR: Complete recanalization
DSMC: Data-safety monitoring committee
DWI: Diffusion weighted imaging
ECASS: European cooperative acute stroke study
ECRI: European cerebrovascular research infrastructure
Eudra CT number: European union drug regulating authorities clinical trials number
EWG: Executive working group
IC/CC ratio: Internal/common carotid ratio
ICU: Intensive care unit
MCA: Middle cerebral artery
mRS: modified Rankin Scale
NASCET: North american symptomatic carotid endarterectomy trial
NIHSS: National institutes of health stroke scale
NINDS: National institute of neurological disorders and stroke
NOR-SASS: Norwegian sonothrombolysis in acute stroke study
NORSTROKE: Norwegian stroke research registry
PAI: Plasminogen activator inhibitor
PAP: Pulmonary arterial pressure
PROBE: Prospective randomised, open-label, blinded endpoint
SAE: Serious adverse events
sICH: symptomatic Intracranial haemorrhage
SITS-MOST: Safe implementation of thrombolysis in stroke monitoring study
SOP: Standard operating procedure
SUSAR: Suspected unexpected serious adverse reaction
TCCS: Transcranial color-coded duplex sonography
TCD: Transcranial doppler
TIBI: Thrombolysis in myocardial infarction
tPA: tissue Plasminogen activator
